# Family history and risk of lung cancer: age-at-diagnosis in cases and first-degree relatives

**DOI:** 10.1038/sj.bjc.6603386

**Published:** 2006-09-26

**Authors:** A Cassidy, J P Myles, S W Duffy, T Liloglou, J K Field

**Affiliations:** 1 Roy Castle Lung Cancer Research Programme, University of Liverpool Cancer Research Centre, 200 London Road, Liverpool L3 9TA, UK; 2 Cancer Research UK Centre for Epidemiology, Mathematics and Statistics, Wolfson Institute of Preventive Medicine, London EC1M 6BQ, UK

**Keywords:** case–control, early onset, familial, genetic, lung cancer

## Abstract

To investigate the little known risk of lung cancer at an early age when a first-degree relative has had such a diagnosis, 579 incident cases and 1157 population controls were studied in Liverpool between 1998 and 2004 using standardised questionnaires covering demography and lifestyle. A history of lung cancer in first-degree relatives was associated with a significantly increased risk in the proband where in both individuals the cancers were diagnosed before the age of 60 years (odds ratio (OR)=4.89; 95% confidence interval (CI): 1.47–16.25). A significantly elevated risk of lung cancer was also observed in association with a relative affected before the age of 60 years, regardless of age-at-onset of the disease (OR=2.08; 95% CI: 1.20–3.59). This finding is strongly consistent with a genetic component in early-onset lung cancer risk.

Smoking is well established as the major aetiological risk factor for lung cancer. Investigators have long hypothesised that individuals differ in their susceptibility to environmental insults and that these differences may be the result of genetic predisposition (Schwartz, 2004). Familial aggregation and increased familial risk for lung cancer have been reported in several studies, providing indirect evidence that genetic factors contribute to susceptibility to lung cancer (Yang *et al*, 1999; Etzel *et al*, 2003; Matakidou *et al*, 2005; Xu *et al*, 2005). Recently, a major lung cancer susceptibility locus was mapped to chromosome 6q23–35 through a genome-wide linkage analysis, further supporting the role of genetic factors in the susceptibility of lung cancer (Bailey-Wilson *et al*, 2004). Biological theory and the experience of other cancers, including breast (Hopper *et al*, 1999), pancreas (James *et al*, 2004) and colon (Strate and Syngal, 2005), suggest that tumours associated with genetic factors tend to occur early in life. Numerous lung cancer studies have investigated the numbers of affected relatives and age-at-onset, with greatest risk seen in families with early-onset lung cancer compared with those whose onset of lung cancer occurred at older ages (Kreuzer *et al*, 1998; Bromen *et al*, 2000; Gauderman and Morrison 2000; Li and Hemminki, 2004; Cote *et al*, 2005). However, information on familial risk by age-at-onset of both the proband and the affected relatives is rare in lung cancer studies. Here we report results for a case–control study that examines age-at-onset in both lung cancer cases and affected relatives.

## MATERIALS AND METHODS

The lung cancer case–control data were derived from an ongoing molecular–epidemiological study of lung cancer in Liverpool, UK, The Liverpool Lung Project (Field *et al*, 2005). Histologically or cytologically confirmed lung cancer cases with primary tumours, resident within the study area, were recruited from participating chest clinics. Population controls were selected from registers of general practitioners in Liverpool and matched to cases by 2-year age group and gender.

A standardised questionnaire was used to determine basic demographic characteristics in addition to details on smoking history, family history of cancer in first-degree relatives and exposure to environmental tobacco smoke (ETS). Lifetime smoking histories were recorded, covering: (i) ever smoked (never-smoker defined as someone who had smoked less than 100 cigarettes in his or her lifetime); (ii) current smoker (yes or no); (iii) duration of smoking (years); (iv) pack-years (calculated from the number of cigarette packs (of 20) smoked per day and years of smoking); and (v) amount smoked (average number of cigarettes per day). Environmental tobacco smoke exposure was defined as being in the presence of a smoker on a regular basis. Exposures to ETS at home, work and in public places (e.g. bars) were recorded separately. Information on history of cancer among first-degree relatives (i.e. parents, brothers and sisters and biological children) were recorded, including age-at-diagnosis, site of cancer and relation to the participant. These data were not validated by death certificate, and smoking information of relatives was not obtained. Individuals were defined as having a history of familial lung cancer if at least one relative with lung cancer was reported. The study protocol was approved by the Liverpool Research Ethic Committee and all research participants provided written, informed consent in accordance with the Declaration of Helsinki Principle.

### Statistical analyses

Significance tests for differences in distributions between cases and controls, odds ratio (OR) estimates of relative risk and 95% confidence intervals (CI) were all estimated using conditional logistic regression. Early- and late-onset lung cancers were defined as before the age of 60 years and at the age of 60 years or later, respectively, in accordance with the average age of lung cancer diagnoses in our study population. To determine whether the risk of developing lung cancer is greater for relatives of cases with early- and late-onset lung cancer, we performed analyses stratified for (i) cases diagnosed before the age of 60 years and at the age of 60 years or later; and (ii) first-degree relatives diagnosed with lung cancer before the age of 60 years and at the age of 60 years or later. Models were adjusted for smoking duration (five categories), ETS exposure at home, work and in public (two categories) and socioeconomic status (six categories). All analyses were performed using STATA.

## RESULTS

Five hundred and seventy-nine incident cases of lung cancer and 1157 population controls were recruited between 1998 and 2004. Overall, the response rate was 58.3% for cases and 61.5% for controls. Table 1 shows the distribution of cases and controls by number of affected relatives, smoking, socioeconomic status and ETS exposure. As expected, the proportion of ever smokers was higher among cases (95.3%) compared with controls (71%), with significant differences observed in terms of duration of smoking (*P*<0.001). The proportion of cases with two or more first-degree relatives was more than twice that of the controls: 5.2 and 2.3%, respectively (*P*=0.03). There were also significant differences between cases and controls in socioeconomic status (*P*<0.001) and exposure to ETS in the home (*P*<0.001). Borderline significant differences were observed for exposure to ETS at work (*P*=0.07).

Although there was a significant trend of increasing risk with numbers of affected relatives (Table 1), there was no significant effect of family history (any *vs* none) of lung cancer in the study population overall or in late-onset cases, regardless of the age of affected relatives. There was, however, a substantial and statistically significant increase in risk where both the lung cancer case and the affected relative were diagnosed with lung cancer before the age of 60 years (OR=4.89; 95% CI: 1.47, 16.25) (*P*=0.01). Significantly elevated ORs were also observed in connection with an affected relative diagnosed before the age of 60 years, regardless of age-at-onset of the case (OR=2.08; 95% CI: 1.20, 3.59) (Table 2). The interaction did not reach formal statistical significance (*P*=0.1).

## DISCUSSION

Our results demonstrate an approximate five-fold increase in risk of lung cancer in individuals aged less than 60 years if first-degree relatives were diagnosed with early-onset lung cancer (i.e. less than 60 years old). We also found a significantly increased risk associated with family history regardless of age-at-onset. The increase in familial risk reported in younger individuals supports the hypothesis that there is a greater likelihood of a genetic component to risk in this group. Previous studies of familial aggregation suggest that family history of lung cancer among first-degree relatives is associated with increased risk for early-onset, but not late-onset, lung cancer. Kreuzer *et al* (1998) reported that lung cancer in a first-degree relative was associated with a 2.6-fold increase in risk of lung cancer of young cases (before 46 years of age), with no elevated risk observed in the older group. Bromen *et al* (2000) reported a 4.75-fold increase in risk of lung cancer among relatives of probands who were diagnosed with lung cancer before the age of 50 years. Li and Hemminki (2004) used a Swedish register of families to estimate standardised incidence ratio for offspring and siblings of cases of lung cancer, and found a substantially increased risk of disease before the age of 50 years in relatives of cases. In an alternative analysis to quantify the lifetime risk of lung cancer in first-degree relatives of early-onset cases diagnosed before the age of 50 years, Cote *et al* (2005) reported a 1.91-fold increased risk of lung cancer for relatives of early-onset cases compared to affected relatives in a control population.

Our results concur with previous reports, but while others have observed age-specific effects in familial lung cancer risk, this is the first study, to our knowledge, demonstrating an increased risk of lung cancer when both the lung cancer case and the affected relative were diagnosed at younger ages. It is difficult for epidemiology to provide conclusive evidence that the accumulation of lung cancer risk has a genetic origin. Cautious interpretation of familial effects on lung cancer risk is therefore required because of the possibility that elevated relative risks are due to shared smoking habits within families (Khoury *et al*, 1988). However, it is difficult to envisage how such confounding could be very strong at young ages and almost nonexistent at older. It is therefore likely that our observed additional risk for early onset disease associated with family history is not due to such confounding. Furthermore, a recent segregation analysis of lung cancer pedigrees, allowing for the effects of smoking sex and age, suggests that multiple genetic factors (possibly multiple genetic loci and interactions) contribute to susceptibility and age-of-onset for lung cancer (Xu *et al*, 2005). The particularly high risk observed in our study associated with early onset in both the proband and the affected relatives adds to the evidence of a genetic component.

In our study, smoking information allowed a detailed adjustment in the analyses. In addition, we controlled for other known environmental factors, such as socioeconomic status and ETS, which have previously been associated with an increased risk of lung cancer. A limitation of the present study pertains to the use of self-reported family history of cancer, which may result in inaccurate risk estimates. Bondy *et al* (1994) evaluated the validity of proband-reported family history of cancer using medical records and death certificates, noting that 85% of probands correctly identified primary lung cancer in first-degree relatives. However, a recent cancer registry study linking individuals to their first-degree family members suggests that in case–control studies of a specific cancer type, cases are more likely than controls to report both true-positive and false-positive family histories of their particular cancer, resulting in inflated estimates of the relative risk (Chang *et al*, 2006). Another limitation of the present study is the possibility of recall bias or other biases in determination of family history in a case–control design (Khoury and Flanders, 1995; Kreuzer *et al*, 1998). Furthermore, because the age-at-onset among relatives was reported by the lung cancer case, inaccuracies may lead to information bias. It is unlikely, however, that such biases could lead to effects as substantial and specific as observed in our study. In conclusion, our study found a substantial and significant increase in risk of having lung cancer before the age of 60 years exclusively associated with a relative having also had lung cancer diagnosed before the age of 60 years. This remains significant after adjustment for smoking, socioeconomic status and ETS exposure and is consistent with a genetic component to risk in early-onset lung cancer.

## Figures and Tables

**Table 1:** Distribution of study-specific characteristics of lung cancer cases and healthy controls, Liverpool, 1998–2004

	**Case**		**Control**		
**Variable**	***N***	**%**	***N***	**%**	***P*-value** *
*Number of lung cancer relatives*					
0	456	78.8	946	81.8	0.03
1	93	16.1	183	15.9	
2	25	4.3	22	1.7	
3	2	0.4	5	0.5	
4	3	0.5	1	0.1	
					
*Smoking (duration)*					
Never	27	4.7	335	29.0	<0.001
1–19 (years)	43	7.4	236	20.4	
20–39 (years)	157	27.1	337	29.1	
40–59 (years)	321	55.4	234	20.2	
⩾60 (years)	31	5.4	15	1.3	
					
*Socioeconomic status* a					
Managerial and professional	79	14.4	270	23.5	<0.001
Intermediate occupations	49	8.9	141	12.3	
Small employers	24	4.4	59	5.1	
Lower supervisory	73	13.3	145	12.6	
Semiroutine and routine	281	51.1	427	37.2	
Long term unemployed	44	8.0	107	9.3	
					
*Home ETS* a					
No	100	17.3	368	31.8	<0.001
Yes	284	49.1	706	61.0	
					
*Work ETS* a					
No	149	25.7	374	32.3	0.07
Yes	246	42.5	776	67.1	
					
*Public ETS* a					
No	81	14.0	286	24.7	0.16
Yes	308	53.2	864	74.7	

ETS=environmental tobacco smoke.

a Numbers do not add up to total due to missing data.

* *P*-values were derived from conditional logistic regression.

**Table 2:** Stratified analyses of early- and late-onset lung cancer in proband and first-degree relatives, Liverpool, 1998–2004

**Age of proband**	**Family history of lung cancer**	**Case**	**Control**	**OR^a^**	**95% CI**	*P*-value*
All ages	No family history	456	946	1.00	Reference	—
	Affected relative <60 years	46	62	2.08	(1.20–3.59)	0.009
	Affected relative ⩾60 years	77	149	1.27	(0.83–1.95)	0.9
						
<60 years	No family history	97	283	1.00	Reference	—
	Affected relative <60 years	15	15	4.89	(1.47–16.25)	0.01
	Affected relative ⩾60 years	14	35	1.37	(0.46–4.01)	0.6
						
⩾60 years	No family history	359	663	1.00	Reference	—
	Affected relative <60 years	31	47	1.46	(0.75–2.86)	0.3
	Affected relative ⩾60 years	63	114	1.10	(0.68–1.78)	0.7

CI=confidence interval; OR=odds ratio.

a ORs adjusted for smoking duration, socioeconomic status and ETS (home, work and public).

* *P*-values were derived from conditional logistic regression.

## References

[bib1] Bailey-Wilson JE, Amos CI, Pinney SM, Petersen GM, de Andrade M, Wiest JS, Fain P, Schwartz AG, You M, Franklin W, Klein C, Gazdar A, Rothschild H, Mandal D, Coons T, Slusser J, Lee J, Gaba C, Kupert E, Perez A, Zhou X, Zeng D, Liu Q, Zhang Q, Seminara D, Minna J, Anderson MW (2004) A major lung cancer susceptibility locus maps to chromosome 6q23–25. Am J Hum Genet 75: 460–474 15272417 10.1086/423857 PMC1182024

[bib2] Bondy ML, Strom SS, Colopy MW, Brown BW, Strong LC (1994) Accuracy of family history of cancer obtained through interviews with relatives of patients with childhood sarcoma. J Clin Epidemiol 47: 89–96 8283198 10.1016/0895-4356(94)90037-x

[bib3] Bromen K, Pohlabeln H, Jahn I, Ahrens W, Jockel KH (2000) Aggregation of lung cancer in families: results from a population-based case–control study in Germany. Am J Epidemiol 52: 497–505 10.1093/aje/152.6.497 10997539

[bib4] Chang ET, Smedby KE, Hjalgrim H, Glimelius B, Adami HO (2006) Reliability of self-reported family history of cancer in a large case–control study of lymphoma. J Natl Cancer Inst 98: 61–68 16391372 10.1093/jnci/djj005

[bib5] Cote ML, Kardia SL, Wenzlaff AS, Ruckdeschel JC, Schwartz AG (2005) Risk of lung cancer among white and black relatives of individuals with early-onset lung cancer. JAMA 293: 3036–3042 15972566 10.1001/jama.293.24.3036

[bib6] Etzel CJ, Amos CI, Spitz MR (2003) Risk for smoking-related cancer among relatives of lung cancer patients. Cancer Res 63: 8531–8535 14679021

[bib7] Field JK, Smith DL, Duffy SW, Cassidy A (2005) The Liverpool Lung Project Research Protocol. Int J Oncol 27: 1633–1645 16273220

[bib8] Gauderman WJ, Morrison JL (2000) Evidence for age-specific genetic relative risks in lung cancer. Am J Epidemiol 151: 41–49 10625172 10.1093/oxfordjournals.aje.a010120

[bib9] Hopper JL, Southey MC, Dite GS, Jolley DJ, Giles GG, McCredie MR, Easton DF, Venter DJ (1999) Population-based estimate of the average age-specific cumulative risk of breast cancer for a defined set of protein-truncating mutations in BRCA1 and BRCA2. Australian breast cancer family study. Cancer Epidemiol Biomarkers Prev 8: 741–747 10498392

[bib10] James TA, Sheldon DG, Rajput A, Kuvshinoff BW, Javle MM, Nava HR, Smith JL, Gibbs JF (2004) Risk factors associated with earlier age of onset in familial pancreatic carcinoma. Cancer 101: 2722–2726 15534880 10.1002/cncr.20700

[bib11] Khoury MJ, Beaty TH, Liang KY (1988) Can familial aggregation of disease be explained by familial aggregation of environmental risk factors? Am J Epidemiol 127: 674–683 3341366 10.1093/oxfordjournals.aje.a114842

[bib12] Khoury MJ, Flanders WD (1995) Bias in using family history as a risk factor in case–control studies of disease. Epidemiology 6: 511–519 8562628 10.1097/00001648-199509000-00009

[bib13] Kreuzer M, Kreienbrock L, Gerken M, Heinrich J, Bruske-Hohlfeld I, Muller KM, Wichmann HE (1998) Risk factors for lung cancer in young adults. Am J Epidemiol 147: 1028–1037 9620046 10.1093/oxfordjournals.aje.a009396

[bib14] Li X, Hemminki K (2004) Inherited predisposition to early-onset lung cancer according to histological type. Int J Cancer 112: 451–457 15382071 10.1002/ijc.20436

[bib15] Matakidou A, Eisen T, Bridle H, O'Brien M, Mutch R, Houlston RS (2005) Case–control study of familial lung cancer risks in UK women. Int J Cancer 116: 445–450 15800943 10.1002/ijc.21012

[bib16] Schwartz AG (2004) Genetic predisposition to lung cancer. Chest 125: 86S–89S 15136430 10.1378/chest.125.5_suppl.86s

[bib17] Strate LL, Syngal S (2005) Hereditary colorectal cancer syndromes. Cancer Causes Control 16: 201–213 15947872 10.1007/s10552-004-3488-4

[bib18] Xu H, Spitz MR, Amos CI, Shete S (2005) Complex segregation analysis reveals a multigene model for lung cancer. Hum Genet 116: 121–127 15599767 10.1007/s00439-004-1212-9

[bib19] Yang P, Schwartz AG, McAllister AE, Swanson GM, Aston CE (1999) Lung cancer risk in families of nonsmoking probands: heterogeneity by age at diagnosis. Genet Epidemiol 17: 253–273 10520209 10.1002/(SICI)1098-2272(199911)17:4<253::AID-GEPI2>3.0.CO;2-K

